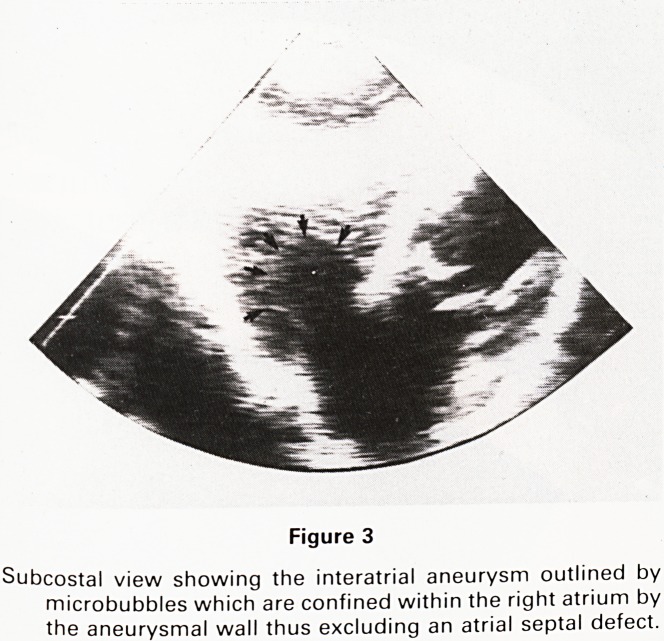# Aneurysm of the Interatrial Septum and Mitral Valve Proplapse—An Aetiological Association?

**Published:** 1988-08

**Authors:** K. S. Channer, R. Bolton, J. Russell Rees, P. Wilde

**Affiliations:** Senior Registrar in Cardiology From the departments of Cardiology and Radiodiagnosis of the Bristol Royal Infirmary, Bristol BS2 8HW; Senior Registrar in Radiology From the departments of Cardiology and Radiodiagnosis of the Bristol Royal Infirmary, Bristol BS2 8HW; Consultant Cardiologist From the departments of Cardiology and Radiodiagnosis of the Bristol Royal Infirmary, Bristol BS2 8HW; Consultant Radiologist From the departments of Cardiology and Radiodiagnosis of the Bristol Royal Infirmary, Bristol BS2 8HW

## Abstract

Mitral valve prolapse is common but aneurysm of the interatrial septum is rare. We report a case in which these two abnormalities of myocardial structure occurred and postulate a common aetiological mechanism.


Bristol Medico-Chirurgical Journal Volume 103 (iii) August 1988

### Aneurysm of the interatrial septum and mitral valve prolapse – an aetiological association?

**Dr K. S. Channer MD MRCP**
Senior Registrar in Cardiology
**Dr R. Bolton FRCS FRCR***
Senior Registrar in Radiology
**Dr J. Russell Rees MD FRCP**
Consultant Cardiologist
**Dr P. Wilde MRCP FRCR**
Consultant Radiologist
From the departments of Cardiology and Radiodiagnosis of the Bristol Royal Infirmary, Bristol BS2 8HW.
*Now Consultant Radiologist, Torbay Hospital, Torquay.

### SUMMARY

Mitral valve prolapse is common but aneurysm of the interatrial septum is rare. We report a case in which these two abnormalities of myocardial structure occurred and postulate a common aetiological mechanism.

### INTRODUCTION

Mitral valve prolapse was first recognised in 1963[1] on cineangiography but it is since the advent of echocardiography that its frequency and associations have been extensively studied. We report a case of aneurysm of the interatrial septum in association with pansystolic mitral valve prolapse and postulate an aetiological association.

### CASE HISTORY

A 73-year-old man presented in July 1985 with a history of increasing breathlessness such that he was dyspnoeic on walking a hundred yards on the flat. He denied chest pain on exertion but complained of a constant dull ache in his left chest and palpitation on exertion. His therapy comprised only of nitrates. On examination he had a sinus bradycardia of 40 beats per minute, and a systolic click and murmur of mitral valve prolapse. He had been seen two years previously with atypical chest pain, and at that time examination was normal and no murmurs were heard.

Full blood count and thyroid function test were normal. A 12 lead electrocardiograph was normal but a 24-hour ambulatory electrocardiograph revealed episodes of atrial bigeminy, paroxysmal atrial fibrillation and sinus bradycardia. He had normal spirometry.

A chest radiograph showed normal lung fields and cardiomegaly (CTR=0.56). A full 2 dimensional, M mode and pulsed Doppler echocardiographic examination was performed. 2D and M mode showed pansystolic mitral valve prolapse (Figure 1). Pulsed Doppler examination of the mitral valve showed obvious systolic turbulence in the left atrium close to the valve leaflets which was diagnostic of mitral regurgitation. The left ventricle was normal in size and contractility and the remaining valves were shown to be normal. The apical four chamber view and the subcostal view both showed aneurysmal bulging of the interatrial septum into the right atrium. The aneurysm lay near the tricuspid orofice but did not appear to obstruct it.

**Figure 1**

M mode echocardiograph of mitral valve showing pansystolic mitral valve prolapse.

**Figure 2**

Subcostal four chamber view showing aneurysm of the interatrial septum (AS), right atrium (RA), left atrium (LA), mitral valve (MV) and left ventricle (LV).

Address for correspondence: Dr K. S. Channer, Department of Cardiology, Bristol Royal Infirmary, Bristol BS2 8HW.

**Figure 3**

Subcostal view showing the interatrial aneurysm outlined by microbubbles which are confined within the right atrium by the aneurysmal wall thus excluding an atrial septal defect.

The intravenous injection of microbubbles in saline allowed clear demarkation of the right side of this structure and free flow to the right ventricle was observed. No passage of microbubbles through the aneurysm to the left atrium was evident. Pulsed Doppler examination of the right side of the aneurysm showed no evidence of an atrial septal defect. Thus the aneurysm although large, was not judged to exhibit any haemodynamic effect.

### DISCUSSION

Mitral valve prolapse is a common finding on echocardiography and up to 5% of the normal population have echocardiographic evidence of it without symptoms or clinical signs[2]. Many of these are young thin women in whom the mitral valve seems excessively large for the ventricle. The frequency of mitral valve prolapse in females decreases with age which argues against a pathological abnormality of the valve. The prognosis is usually good in these cases[3]. Conversely, clinical mitral valve prolapse which is characterised by a systolic click and late systolic murmur, is more common in middle age and elderly men and may progress to severe mitral regurgitation. This clinical subgroup is at increased risk of endocarditis[4] and embolic phenomena[5,6]. Mitral valve prolapse is now the commonest cause for mitral valve replacement in the middle aged and elderly[7,8]. Many patients with mitral valve prolapse as demonstrated in our case, experience atypical chest pain[3] and ventricular and supraventricular arrhythmias[9] which are thought to originate from abnormal tensions on the papillary muscles.

Pathologically prolapsing mitral valves show myxomatous degeneration[10,11] and studies have shown them to have abnormal collagen fibres. There remains controversy however, as to whether the degeneration is the cause or the result of the prolapse[3]. The association of mitral valve prolapse with inherited disorders of connective tissue e.g. Marfans syndrome, suggest that the degeneration may be the primary event. The case described reports the association of aneurysmal bulging of the interatrial septum in association with mitral valve prolapse. Although mitral valve prolapse is common, aneurysm of the interatrial septum is very rare and is usually associated with congenital valve lesions which cause very high intra atrial pressures. There have been two previous reports of this association. A series of 5 patients[12] were reported from Italy in whom mitral valve prolapse and tricuspid valve prolapse (in 4) were associated with abnormal bulging of the interatrial septum. Three had aneurysmal bulging and in 2 only localised portions of the septum were seen to be abnormal. These patients were younger than our case. In the other report of two cases[13] the interatrial aneurysm was found incidentally at postmortem examination. The tissues were not studied histologically for connective tissue abnormalities.

We believe these cases provide evidence in favour of a generalised collagen disorder affecting the intracardiac structures. Our case also illustrates the role of modern echocardiographic imaging in elucidating the condition which is par excellence, a new syndrome of modern technology.

### REFERENCES

1. BARLOW, J. B., POCOCK, W. A., MARCHAND, P., DENNY, M. (1963) The significant late systolic murmurs. *Am.Heart J.* **66**, 443–452.
2. SAVAGE, D. D., GARRISON, R. J., DEVEREUX, R. B., CASTELLI, W. P., ANDERSON, S. J., LEVY, D., MCNAMARA, P. M., STOKES, J., KANNEL, W. B., FEINLEIB, M. (1983) Mitral valve prolapse in the general population. 1. Epidemiological features. The Framingham study. *Am.Heart J.* **106**, 571–577.
3. OAKLEY, C. M. (1985) Mitral valve prolapse. *Quart J Med* **56**, 317–320.
4. CLEMENS, J. D., HORWITZ, R. I., JAFFE, C. C., FEINSTEIN, A. R., STANTON, B. F. (1982) A controlled evaluation of the risk of bacterial endocarditis in persons with mitral valve prolapse. *N Engl J Med* **307**, 776–781.
5. WILSON, L. A., KEELING, P. W. N., MALCOLM, A. D., RUSSELL, R. W. R. (1977) Visual complications of mitral valve prolapse. *Br Heart J* **42**, 86–88.
6. KOSTUK, W. J., BOUGHNER, D. R., BARNETT, H. J. M., SILVER, M. D. (1977) Strokes: A complication of mitral leaflet prolapse. *Lancet* **ii**, 313–316.
7. GUY, F. C., MACDONALD, R. P. R., FRASER, D. B., SMITH, E. R. (1980) Mitral valve prolapse as a cause of hemodynamically important mitral regurgitation. *Can J Surg* **23**, 166–170.
8. WALLER, B. F., MORROW, A. G., MARON, B. J., DEL NEGRO, A. A., KENT, K. M., MCGRATH, F. J., WALLACE, R. B., MCINTOSH, M. D., ROBERTS, W. C. (1982) Etiology of clinically isolated severe chronic pure mitral regurgitation. Analysis of 97 patients over 30 years of age having mitral valve replacement. *Am.Heart J.* **104**, 276–288.
9. DEMARIA, A. N., AMSTERDAM, E. A., VISMARA, L. A., NEUMANN, A., MASON, D. T. (1976) Arrythmias in the mitral valve prolapse syndrome. Prevalence, nature and frequency. *Ann Intern Med* **84**, 656–660.
10. DAVIES, M. J., MOORE, B. P. BRAIMBRIDGE, M. V. (1979) The floppy mitral valve. Study of the incidence, pathology and complications in surgical, necropsy and forensic material. *Br Heart J* **40**, 468–481.
11. OLSON, E. G. J., AL-RUFAIE, H. K. (1980) The floppy mitral, valve: study on pathogenesis. *Br Heart J* **44**, 674–683.
12. ILCETO, S., PAPA, A., SORINO, M., RIZZON, P. (1984) Combined atrial septal aneurysm and mitral valve prolapse: detection by two dimensional echocardiography. *Am J Cardiol* **54**, 1151–1153.
13. ROBERTS, W. C. (1984) Aneurysm (redundancy) of the atrial septum (fossa ovale membrane) and prolapse (redundancy) of the mitral valve. *Am J Cardiol* **54**, 1153–1154.

## Figures and Tables

**Figure 1 f1:**
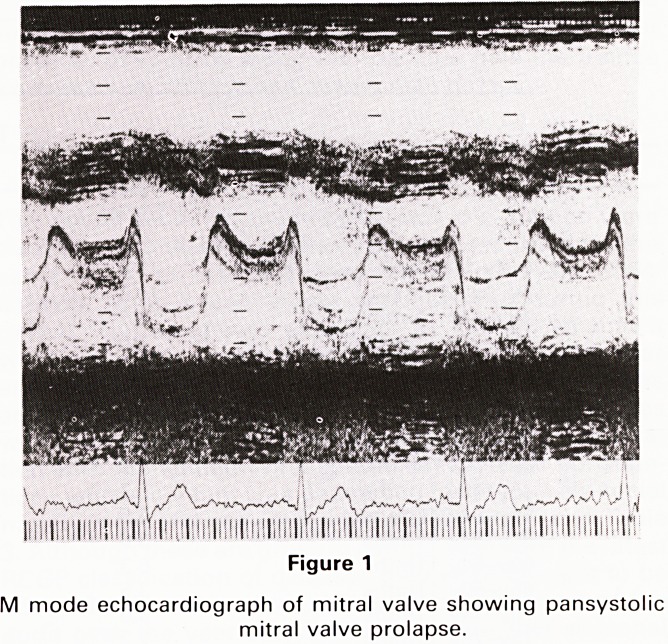


**Figure 2 f2:**
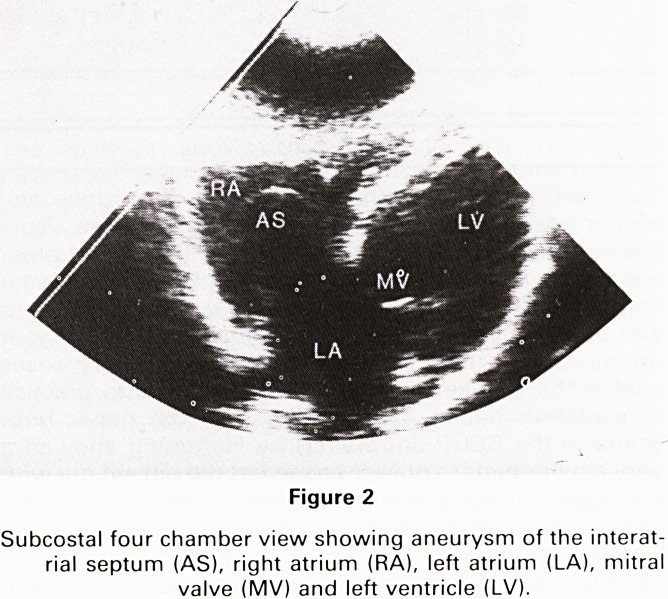


**Figure 3 f3:**